# How Ben Butler Got Rich

**Published:** 1887-12

**Authors:** 


### HOW BEN. BUTLER GOT RICH.

### Young Men of To-day May do Likewise if They Follow Advice Given.

General B. F. Butler being asked for some suggestions on gaining success, stated that when he was a young lawyer, practicing in Lowell, Mass., a bank president advised him to take his little deposit and buy real estate from which he could be deriving some revenue. The general said that he had but little money and was uncertain as to his future.

"Never mind," said the bank president, "go to the next public auction of real estate, bid off a lot with a building of some kind on it, pay down what money you have and give your promissory notes for the balance. You will come out all right."

General Butler says this advice was good. When a man has obligated himself, by his notes, to pay money at a certain time, it inclines him to economy. He followed the advice, and in time became the owner of several parcels of valuable real estate in Lowell.

Two classes will not be likely to heed such advice—the improvident and the over- cautious. The latter will be apt to say: "It would be all right but for those dreadful promissory notes. They are always running on and if a man falls sick they do not wait for him to get well."

There is this danger, of course, but one can make no business venture without some risk, and with the knowledge acquired by recent investigations of the cause of most ordinary ailments, and the means of cure, one runs little risk from that source. It is now known that most of the common ailments have their origin in deranged kidneys. They are the chief blood purifiers of the system and when disordered a breaking down somewhere is soon inevitable, because the poison, which in their healthy condition is eliminated, is carried through the entire system.

Put them in order and health returns.

C. D. Dewey, a successful man, president of the Johnston Harvester Company, Batavia, N. Y., gives his experience as follows:

In 1882 my health was failing, my head pained me constantly, my appetite was uncertain, I could not sleep soundly. I attributed this to the extreme pressure of business cares, but I grew worse, and finally was confined to my bed for two months It seemed as though I would "never recover" my former health. Under the aid of stimulants I gradually gained strength, so that in a few months I was able to attend to business, but I could walk only with the assistance of a cane, and then in a slow and unsteady manner. I continued somewhat in the same condition until February last, when I used Warner's safe cure. It has cured me. I consider it a valuable remedy and can highly recommend it.

Young men have but to use ordinary prudence, and when any derangement occurs if they use the same means as did this successful business man, they may feel a constant assurance of their ability to carry to successful conclusion all ordinary business projects, including the care of their promissory notes when due.

